# Impaired intracortical inhibition demonstrated in vivo in people with Dravet syndrome

**DOI:** 10.1212/WNL.0000000000003868

**Published:** 2017-04-25

**Authors:** William M. Stern, Josemir W. Sander, John C. Rothwell, Sanjay M. Sisodiya

**Affiliations:** From the Department of Clinical and Experimental Epilepsy, NIHR University College London Hospitals Biomedical Research Centre (W.M.S., J.W.S., S.M.S.), and Sobell Department of Motor Neuroscience and Movement Disorders (J.C.R.), UCL Institute of Neurology; Epilepsy Society (W.M.S., J.W.S., S.M.S.), Chalfont St Peter, UK; and Stichting Epilepsie Instellingen Nederland (SEIN) (J.W.S.), Heemstede, the Netherlands.

## Abstract

**Objective:**

Dravet syndrome is a rare neurodevelopmental disorder characterized by seizures and other neurologic problems. *SCN1A* mutations account for ∼80% of cases. Animal studies have implicated mutation-related dysregulated cortical inhibitory networks in its pathophysiology. We investigated such networks in people with the condition.

**Methods:**

Transcranial magnetic stimulation using single and paired pulse paradigms was applied to people with Dravet syndrome and to 2 control groups to study motor cortex excitability.

**Results:**

Short interval intracortical inhibition (SICI), which measures GABAergic inhibitory network behavior, was undetectable in Dravet syndrome, but detectable in all controls. Other paradigms, including those testing excitatory networks, showed no difference between Dravet and control groups.

**Conclusions:**

There were marked differences in inhibitory networks, detected using SICI paradigms, while other inhibitory and excitatory paradigms yielded normal results. These human data showing reduced GABAergic inhibition in vivo in people with Dravet syndrome support established animal models.

Dravet syndrome (DS; OMIM #607208) is an epileptic encephalopathy, characterized by seizures that are often resistant to treatment, and onset is typically with complex febrile seizures, usually in the first year of life. Substantial developmental delay is a common feature; further problems, such as gait disorder, feeding or appetite difficulties, behavioral problems, and sleep disturbance, also occur. Development is normal prior to seizure onset.^1,2^ Structural brain imaging is usually normal, but various structural abnormalities have been described, including focal brain atrophy, cortical dysplasia, and hippocampal sclerosis.^3^ EEG may be normal at seizure onset, but later typically shows multifocal epileptiform abnormalities and a disturbed background rhythm.^4^

Mutations in *SCN1A*, encoding the α subunit of a voltage-gated sodium channel (Na_v_1.1), have been found in >80% of affected individuals; the majority of these mutations arise de novo,^5^ and the *SCN1A* variants include both missense and truncating mutations in addition to whole gene deletion, suggesting a loss of function mechanism.^6^

The mechanisms by which *SCN1A* mutations cause the features of DS have been investigated using knockout mouse models (*Scn1a*^+/−^ and *Scn1a*^−/−^). Homozygous *Scn1a*^−/−^ mice had seizures and ataxia, leading to death by day 15, while heterozygous *Scn1a*^+/−^ mice showed spontaneous seizures, but some survived past 15 weeks.^7^ Whole-cell sodium current recordings from mouse neurons suggest that DS-causing mutations do not affect sodium currents in excitatory pyramidal cells, a finding that may be explained by upregulation of other sodium channels, such as Na_v_1.3. Recordings from GABAergic inhibitory interneurons using the same mouse models show reduced sodium currents.^7,8^ This leads to an increased threshold for action potential generation in affected inhibitory cells.^6^ It has been suggested that DS-causing *SCN1A* mutations reduce the activity of cortical inhibitory networks, with the resultant imbalance of neuronal activity leading to seizures. Apart from seizures, *Scn1a*^+/−^ mice exhibit behavioral problems such as hyperactivity, cognitive difficulties, and impaired social interaction. These may be attributable to reduced GABAergic inhibition in frontal cortical networks.^9^ A recent in vivo study of inhibitory interneurons in the same mouse model of DS, however, demonstrated normal spontaneous firing patterns, suggesting that the picture may be more complex.^10^

While GABAergic inhibition has been well-studied in mouse models of DS, little has been done to understand how inhibitory networks are affected in humans with DS. An EEG-fMRI study of 10 people with DS could not identify a common network underlying epileptiform discharges.^11^

In the current study, transcranial magnetic stimulation (TMS) was used to probe cortical excitability in people with DS. TMS can be used to stimulate motor cortex noninvasively, triggering a motor evoked potential (MEP) in a target muscle. The resting motor threshold (rMT) measures the ease of activation of motor cortex via excitatory networks, while paired pulse paradigms with variable interstimulus intervals test inhibitory and excitatory intracortical circuitry.

We hypothesized that GABAergic inhibition would be reduced in people with DS compared to control groups. GABAergic inhibition can be measured using inhibitory paired pulse paradigms such as short interval intracortical inhibition (SICI) and long interval intracortical inhibition (LICI).^12^ We also hypothesized that rMT and intracortical facilitation (ICF), which are less influenced by GABAergic inhibition, would be normal in DS.

## METHODS

### Standard protocol approvals, registrations, and patient consents.

The project was approved by the local ethical review board (National Research Ethics Service Committee London: Camden and Islington); TMS parameters were within current safety guidelines.^13^ A safety questionnaire was completed for each participant. Participants gave written informed consent. In those lacking capacity, a relative was asked to give assent.

People with DS were identified using an existing clinical registry. Inclusion criteria for people with DS were as follows: clinical criteria for diagnosing DS (normal developmental skills before onset; generalized, or unilateral, or alternating unilateral hemiclonic, febrile, and afebrile seizures beginning in the first year of life, with subsequent myoclonic and generalized tonic-clonic seizures and, sometimes, partial seizures; absence of epileptiform EEG discharges in the initial EEG studies, if available, with later generalized spike-wave and polyspike-wave discharges, focal abnormalities, and possible early photosensitivity; evidence of delayed development from the second year of life onward^1,14^) and the presence of an *SCN1A* mutation confirmed in a clinically accredited laboratory.

Six people with DS were recruited. Five had full TMS testing and 1 only tolerated part of the protocol. Control data were gathered from 10 people with non-DS epilepsy taking antiepileptic medication and 10 healthy participants.

The technical details of the hardware and software used in this study have been published, as have the techniques used to identify the motor hot spot for the adductor pollicis brevis (APB) muscle.^15^

The following paradigms were tested: rMT, SICI, ICF, and LICI. Exact definitions and experimental details of the paradigms used have been published.^15^ The following interstimulus intervals were used: for SICI, 2 and 5 ms; for ICF, 10 and 15 ms; for LICI, 100, 150, 200, and 250 ms.

rMT is a measure of motor cortex excitability, with high rMT implying low excitability.^12^ SICI is mediated by GABA_A_ergic intracortical circuits^16^ while ICF is mediated by glutamatergic intracortical circuits, possibly alongside a reduction in GABAergic inhibition.^12^ LICI is mediated by GABA_B_ergic intracortical circuits.^12^

### Statistical analysis.

rMT for cases and the 2 control groups was compared using univariate analysis of variance (ANOVA). SICI, ICF, and LICI results for cases and controls were compared using multivariate ANOVA for each paradigm. *p* Values of 0.05 or lower were interpreted as statistically significant. Post hoc analysis was performed using Tukey honest significant difference (HSD) test. All analysis was performed using IBM (Armonk, NY) SPSS Statistics, version 22.0.

## RESULTS

### Participants.

Six adults with typical DS were recruited (D1–D6); 5 completed all TMS paradigms, while the 6th subject was unwilling to continue after initial measurement of rMT. All participants with DS had a known mutation in *SCN1A* (table 1). Control participants included 10 consecutive people with treated non-DS epilepsy (E1–E10) and 10 healthy controls (H1–H10). Our healthy control data have been reported.^15^

**Table 1:**
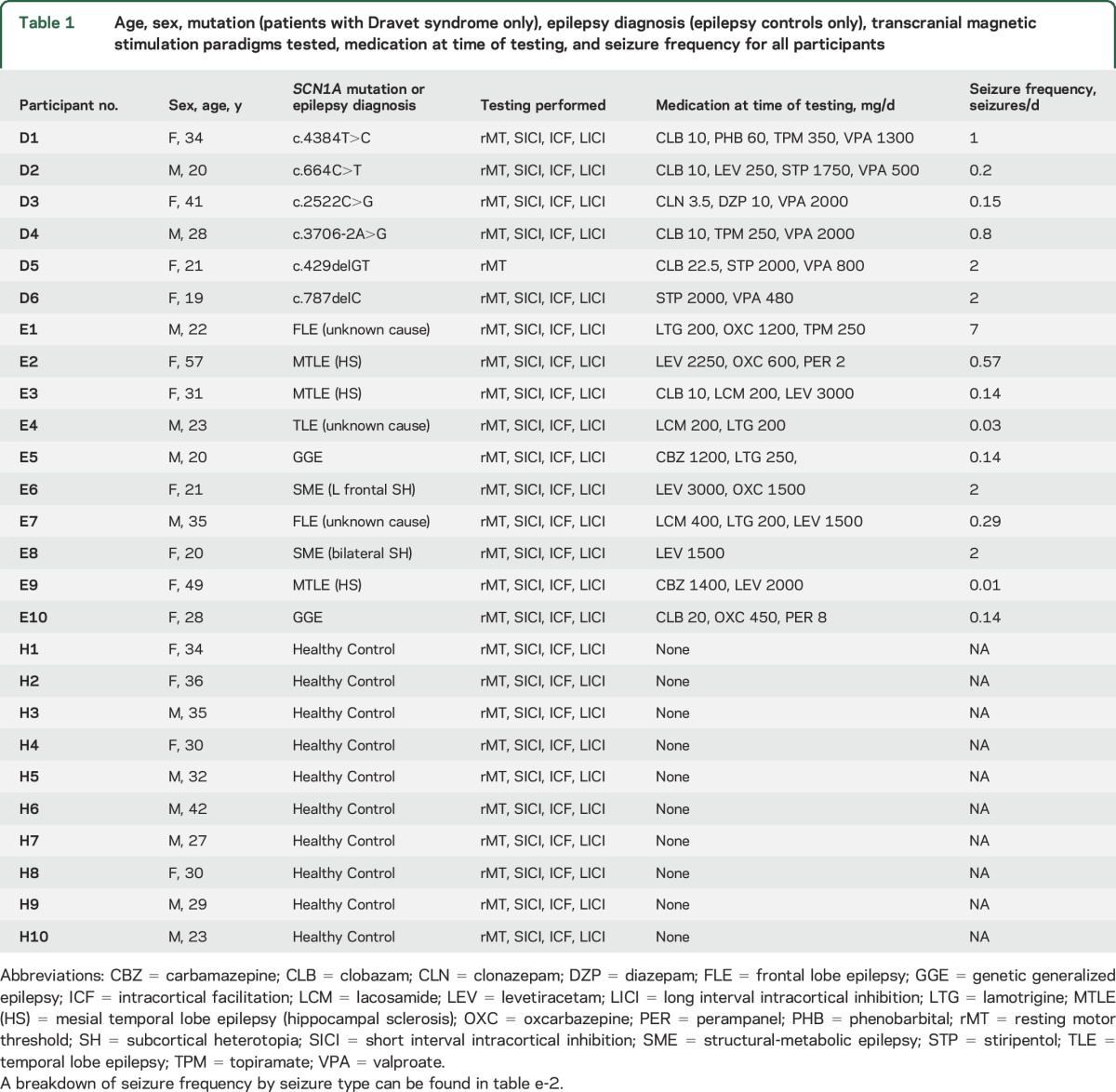
Age, sex, mutation (patients with Dravet syndrome only), epilepsy diagnosis (epilepsy controls only), transcranial magnetic stimulation paradigms tested, medication at time of testing, and seizure frequency for all participants

Table 1 shows demographic information, genetic diagnosis (for those with DS), epilepsy diagnosis (for epilepsy controls), medication at time of testing, and seizure frequency. There were no significant differences in age or sex among the 3 groups (age: univariate ANOVA *p* = 0.61; sex: Fisher exact test *p* = 0.68).

### Medication and seizure frequency.

Those with DS were taking a mean of 3.2 medications each, while the epilepsy controls were taking a mean of 2.5 medications each (table 1); there was no significant difference (unpaired, 2-tailed *t* test, *p* = 0.14). Drugs used in the 2 groups do not match exactly; 3/6 with DS were taking stiripentol, which is rarely used outside of DS, while some epilepsy controls were taking drugs such as carbamazepine and lamotrigine, which are typically avoided in DS. Mean reported seizure frequency in the DS group was 1.3 seizures per day, while epilepsy controls reported a mean of 1.0 seizure per day; there was no significant difference (unpaired, 2-tailed *t* test, *p* = 0.76). Healthy controls were taking no medication, and reported no seizures.

### Resting motor threshold.

**Figure 1:**
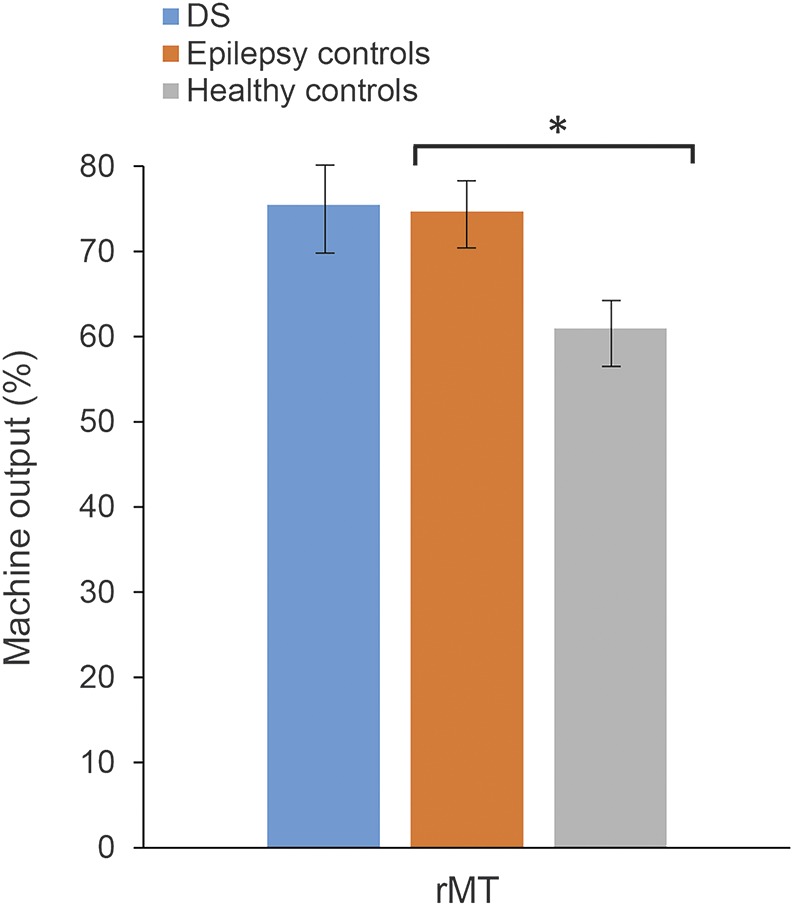
Resting motor threshold (rMT) in all groups Mean results for rMT for people with Dravet syndrome (DS), epilepsy controls, and healthy controls. There was a significant difference (indicated by *) between healthy controls and epilepsy controls (univariate analysis of variance and post hoc Tukey honest significant difference test; *p* = 0.04), but no significant difference between DS and either control group. Error bars show standard error.

rMT was measured in 6 people with DS. Average rMT was 74.7% of maximum machine output, SD 10.8%, range 55%–85% (figure 1). This average and range is similar to that seen in epilepsy controls (75.5%, SD 12.9%, range 59%–91%; unpaired, 2-tailed *t* test *p* = 0.90), implying similar baseline cortical excitability in the groups. Meaningful comparison between epilepsy groups and healthy controls is difficult, since antiepileptic medication is known to affect rMT. A univariate ANOVA comparing rMT in all 3 groups showed significant differences (*p* = 0.03); post hoc analysis confirmed a significant difference only between epilepsy controls and healthy controls (Tukey HSD test; *p* = 0.04).

### Short interval intracortical inhibition.

**Figure 2:**
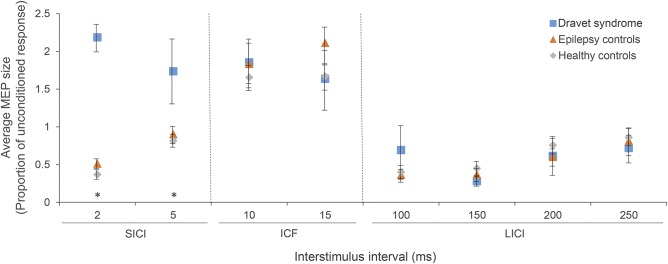
Short interval intracortical inhibition (SICI), intracortical facilitation (ICF), and long interval intracortical inhibition (LICI) in all groups Mean results for SICI, ICF, and LICI for people with Dravet syndrome (DS), epilepsy controls, and healthy controls. There was a significant difference (indicated by *) between DS and both control groups in SICI only. Error bars show standard error. MEP = motor evoked potential.

SICI paradigms using ISI of 2 and 5 ms did not cause inhibition in our DS group. Instead, there was significant facilitation, with mean MEP responses of 2.18 (SD 0.47) at 2 ms and 1.74 (SD 1.03) at 5 ms normalized to unconditioned responses. By contrast, all 20 controls, including those with and without epilepsy, showed inhibition at an ISI of 2 ms (mean normalized response 0.44, SD 0.15), and most also showed inhibition at an ISI of 5 ms (mean normalized response 0.86, SD 0.32). Data for SICI across DS and control groups are shown in figure 2. A multivariate ANOVA comparing SICI at both ISIs in DS to all controls showed a significant difference (*p* < 0.0001). Post hoc analysis with adjustment for multiple comparisons (Tukey HSD test) confirmed a significant difference between DS and each control group at both ISIs (2 ms: DS vs epilepsy controls, *p* < 0.0001; DS vs healthy controls, *p* < 0.0001; 5 ms: DS vs epilepsy controls, *p* = 0.02; DS vs healthy controls, *p* = 0.01). There was no significant difference between non-DS epilepsy controls and healthy controls at either ISI (2 ms, *p* = 0.38; 5 ms, *p* = 0.92).

There was no significant difference in ICF or LICI between any group (figure 2) (multivariate ANOVA, ICF *p* = 0.58, LICI *p* = 0.65).

## DISCUSSION

Our key finding is the lack of GABA-mediated SICI in people with DS, consistent with our main hypothesis. There was no inhibition at 2 ms ISI in any people with DS, while inhibition was detected in all 20 controls; the difference was significant. There was also significantly less inhibition in the DS group at 5 ms ISI.

Instead of the expected normal inhibition of the MEP at ISIs of 2 and 5 ms, the DS group showed facilitation. In healthy participants, a subthreshold conditioning stimulus causes some activity in both excitatory intracortical and inhibitory intracortical neurons. In controls, as in the extensive TMS literature on paired pulse stimulation, the inhibitory effects were dominant at shorter ISIs (SICI) while the excitatory effects were dominant at longer ISIs (intracortical facilitation). In our DS group, the excitatory effects appeared to be dominant at shorter ISIs. This may be because the subthreshold conditioning stimulus failed to activate a sufficient number of inhibitory interneurons to trigger detectable inhibition.

LICI, which is believed to be mediated by the same GABAergic interneurons, was robustly detectable in all groups, with no difference detected between DS and controls.

Aside from a different ISI, an obvious difference between SICI and LICI paradigms is that the conditioning pulse in SICI is given at subthreshold intensity, while that in LICI is given at suprathreshold intensity.

There is a striking parallel between our findings in people with DS and the data from mouse models of the condition. *Scn1a*^+/−^ mouse inhibitory interneurons showed a reduction in sodium currents, leading to an increased threshold for action potential generation in these cells^6–8^; subthreshold conditioning stimuli in our SICI paradigm failed to generate any detectable inhibition on subsequent MEPs in people with DS. Suprathreshold stimuli were still able to generate action potentials in *Scn1a*^+/−^ mouse inhibitory interneurons; the suprathreshold conditioning stimuli in LICI triggered an inhibitory effect on the MEP similar to that seen in controls.

The presence of normal LICI suggests that inhibitory cortical networks are functionally intact in people with DS, as indeed they are in the *Scn1a*^+/−^ mouse.^10^ The lack of SICI, however, suggests that these inhibitory networks may show reduced sensitivity.

It is worth noting that a TMS impulse activates axons directly, while physiologic activation of the same neurons would occur synaptically. The spreading depolarization ahead of a synaptically triggered action potential is much larger than needed to discharge the membrane and therefore might be relatively insensitive to small changes in membrane threshold. Thus, the relative insensitivity of inhibitory neurons to subthreshold magnetic stimuli in DS may not imply an equally significant reduction in the physiologic firing of these neurons. A pattern of reduced sodium channel function but relatively preserved spontaneous firing patterns has been shown in animal models.^7,10^

The fact that our patients with DS were all on antiepileptic drug (AED) treatment implies that the therapies used did not fully neutralize the pathologic effects of the *SCN1A* mutation. This is consistent with their clinical presentation; none of our DS group members was seizure-free.

There are other potential explanations for the lack of SICI and the presence of LICI in DS. SICI is believed to be mediated via GABA_A_ receptors on corticospinal neurons, while LICI is mediated via GABA_B_ receptors.^16^ It is possible that expression or activity of these receptors might be affected unequally in DS, such that GABA_B_-mediated inhibition functions normally and GABA_A_-mediated inhibition does not. Another possibility is that the longer ISIs in LICI paradigms are important; for example, the *SCN1A* mutation might delay release of GABA from the inhibitory cortical neurons, thus preventing inhibition at the shorter ISIs of SICI. A further explanation would be that the mechanisms controlling SICI are normal in people with DS, but the inhibition is not detectable as the *SCN1A* mutation leads to extra facilitation not found in controls. Facilitation can be found at short ISIs in healthy people, a process termed short ICF, but this is typically triggered by a suprathreshold conditioning stimulus.^17^ ICF uses a subthreshold stimulus, and was similar in DS and controls in our study. None of these alternative explanations is supported by the animal data.

rMT was lower in healthy controls compared to either the DS group or epilepsy controls, although the comparison between DS and healthy controls did not reach statistical significance. The difference is most likely due to effects of the AEDs used by our DS and epilepsy populations. There was no difference in rMT between the DS group and epilepsy controls, who were on a similar number of medications. Likewise, ICF was similar in DS and controls. These findings are in keeping with our hypothesis that DS would not affect rMT or ICF.

AEDs are known to modify TMS results, with different drugs affecting different measures. Our inclusion of a control group with epilepsy is helpful in this regard, as it demonstrates that SICI is robustly detectable in people with epilepsy of various types despite long-term AED treatment, which aids a meaningful comparison between cases and controls. It was not possible to match exactly the AED exposure of our patients with DS and epilepsy controls. Sodium channel-blocking AEDs, including commonly used AEDs like lamotrigine and carbamazepine, may exacerbate DS; they are typically avoided in this population. Conversely, stiripentol is in common use in DS, but is rarely used to treat other types of epilepsy. Given the impossibility of matching drugs exactly, we instead compared the number of AEDs. Moreover, although sodium channel blocking AEDs are widely recognized to increase rMT, they do not affect SICI.^18^ We consider that disparities between drug types cannot explain the unusual pattern of SICI in our DS group.

People with DS commonly have substantial learning difficulties and may have other behavioral problems; performing TMS can be challenging in this population. However, this seems an unlikely explanation for the robust differences seen in SICI between our DS and control groups, since other TMS paired pulse measurements (ICF and LICI) were no different vs controls. We have previously reported TMS results in people with alternating hemiplegia of childhood, a neurodevelopmental disorder associated with learning and behavioral difficulties of comparable severity to DS; this group had normal SICI,^15^ supporting the specificity of our results for DS.

Our DS group is smaller than our control groups. DS is a rare condition, and many people with DS are not able to undergo TMS testing, due to learning and behavioral difficulties. People with a more severe phenotype were not approached for this study, since it was believed unlikely that they would tolerate testing; this is a potential bias (table e-1 at Neurology.org). All our participants are adults, in whom the electrophysiologic profile may differ from that in children.^19^ Only small numbers of people with DS could be recruited. The differences in SICI between the DS and control groups are statistically significant, but need cautious interpretation and replication.

We used TMS to study motor cortex excitatory and inhibitory networks in people with DS. There were clear abnormalities in inhibitory networks, detected using SICI paradigms, while other inhibitory and excitatory paradigms yielded normal results. These human data show reduced GABAergic inhibition in vivo in DS, supporting established animal models. The specific absence of SICI, in the presence of normal LICI, suggests that inhibitory networks in people with DS are functionally intact, but have a reduced sensitivity to low-intensity stimuli. TMS measures may provide rapid-readout biomarkers for cortical excitability and its modulation by treatment in DS.

## Supplementary Material

Data Supplement

## GLOSSARY

AED: antiepileptic drug
ANOVA: analysis of variance
APB: adductor pollicis brevis
DS: Dravet syndrome
HSD: honest significant difference
ICF: intracortical facilitation
LICI: long interval intracortical inhibition
MEP: motor evoked potential
rMT: resting motor threshold
SICI: short interval intracortical inhibition
TMS: transcranial magnetic stimulation
