# Supplementary material for: Impaired intracortical inhibition demonstrated in vivo in people with Dravet syndrome
Source: Neurology. 2017 Apr 25;88(17):1659–65. doi: 10.1212/WNL.0000000000003868 (PMC5405762; doi:10.1212/WNL.0000000000003868)
Supplement: Data Supplement [file supp_88_17_1659__index.html]

Impaired intracortical inhibition demonstrated in vivo in people with Dravet syndrome — Data Supplement 

# Impaired intracortical inhibition demonstrated in vivo in people with Dravet syndrome

## Data Supplement

**Neurology® data supplements are not copyedited before publication. Published editorials and translations have been copyedited.  
 © 2017 American Academy of Neurology.  
  
 Files in this Data Supplement:**

- e-Tables - PDF
