# Supplementary material for: Impaired intracortical inhibition demonstrated in vivo in people with Dravet syndrome
Source: Neurology. 2017 Apr 25;88(17):1659–65. doi: 10.1212/WNL.0000000000003868 (PMC5405762; doi:10.1212/WNL.0000000000003868)
Supplement: Data Supplement [file supp_WNL.0000000000003868_e-tables.pdf]

Subject number	Sex, Age	*SCN1A* mutation	Age at first seizure	First seizure while febrile	Learning difficulties	EEG	MRI	Seizure types	Additional information
D1	F, 34	c.4384T>C; reported in association with Dravet Syndrome, and described in detail in Catarino et al.* (case 11)	7 months	Yes	Severe	Multifocal spike and wave discharges with frequent seizures	Normal study	GTCS, TS	Prolonged lateralised seizures at 8 months. Detailed description of phenotype in Catarino et al.* (Case 11)
D2	M, 20	c.664C>T de novo	6 months	Yes	Severe	Multifocal spikes (interictal record only)	Not available	GTCS	Prolonged lateralised seizures, myoclonic jerks
D3	F, 41	c.2522C>G	8 months	Yes	Severe	Global dysfunction without epileptiform activity	No gross abnormality	GTCS, TS	Prolonged hemiclonic febrile seizure at onset, recurrent febrile and afebrile seizures, delayed development after seizure onset. Gait and behavioural difficulties.
D4	M, 28	c.3706-2A>G; leading to aberrant splicing	5 months	Yes	Severe	Multifocal sharp waves and multiple seizures recorded	Left hippocampal sclerosis	GTCS, TS	30 minute lateralised seizure at onset, development normal at 6 months but then delayed. Described in Catarino et al.* (Case 21).
D5	F, 21	c.429delGT; frameshift, generating premature stop codon leading to premature protein truncation	4 months	Yes	Moderate	Independent left and right sided spike and wave, epileptiform abnormalities in sleep	No gross abnormality	GTCS, MJ	Worse seizure control with sodium blockers. Typical crouched gait.
D6	F, 19	c.787delC; de novo	4 months	Yes	Moderate	Multiple seizures captured with possible right centroparietal onset	No gross abnormality	GTCS	Seizure onset at 4 months with 90 minute generalised seizure

Supplementary table e-1: Phenotypic information for people with Dravet Syndrome. Abbreviations: GTCS = generalised tonic clonic seizure, TS = tonic seizure, MJ = myoclonic jerk.

*Catarino et al. ‘Dravet syndrome as epileptic encephalopathy: evidence from long-term course and neuropathology’, Brain 2011 Oct;134(Pt 10):2982-3010

Subject number	*Seizure types and frequency*
D1	Two generalised tonic-clonic seizures per week, five motor seizures per week with loss of awareness
D2	One to two generalised tonic-clonic seizures per month
D3	One generalised tonic-clonic seizure per month, daily motor seizures with loss of awareness
D4	Two generalised tonic-clonic seizure per week, three motor seizures per week with loss of awareness
D5	Two generalised tonic-clonic seizures per week, frequent myoclonus
D6	Four generalised tonic-clonic seizures per week
Subject number	
E1	Five to ten focal motor seizures daily
E2	One focal aware seizure per week, one focal unaware seizure per month
E3	Five focal unaware seizures per month, one generalised tonic-clonic seizure per month
E4	One generalised tonic-clonic seizure per month
E5	One generalised tonic-clonic seizure per week
E6	One generalised tonic-clonic seizure per month, two focal unaware seizures per day
E7	Five generalised tonic-clonic seizures per year, three focal unaware seizures per week
E8	Two focal unaware seizures per day
E9	One focal unaware seizure per year
E10	One generalised tonic-clonic seizure per month, one absence seizure per week

Supplementary table e-2: Seizure types and frequency for each subject with epilepsy
